# The increasing role of the allergist in the management of infusion reactions at the Oncology Infusion Center

**DOI:** 10.3389/falgy.2024.1479469

**Published:** 2024-12-11

**Authors:** Julián Borrás Cuartero, Maria Cruz Torres Górriz, Cristina Isabel Stein Coronado, Adrián Germán Sánchez, Cristina Giovanna Pesántez Méndez, Maria Dolores Latorre Ibáñez, Ernesto Enrique

**Affiliations:** ^1^ Allergy Department, Castellon Provincial Hospital Consortium, Castellon de la Plana, Spain; ^2^ Allergy Department, Castellon University General Hospital, Castellon de la Plana, Spain; ^3^ Doctoral School, Jaume I University, Castellon de la Plana, Spain; ^4^ FISABIO Foundation Research Group, Valencia, Spain; ^5^ RIA Laboratory Service - Radiopharmacy, Castellon Provincial Hospital Consortium, Castellon de la Plana, Spain

**Keywords:** drug allergy, Rapid Drug Desensitization, Same-Day Desensitization, chemotherapy allergy, personalized and precision medicine

## Abstract

**Background:**

Hypersensitivity reactions to chemotherapy disrupt treatment schedules and compromise patient outcomes. Rapid Drug Desensitization (RDD) enables patients to tolerate future treatments after an allergy workup. However, Same-Day Desensitization (SDD) is a novel approach that capitalizes on RDD to allow the continuation of chemotherapy on the same day as the index reaction, preventing treatment delays.

**Objective:**

To evaluate the safety and efficacy of SDD in managing hypersensitivity reactions during chemotherapy and emphasize the essential role of allergists in the Oncology Infusion Center (OIC) for accurate drugs hypersensitivity reactions (DHRs) phenotyping and management.

**Methods:**

This retrospective cohort included patients experiencing DHRs during chemotherapy. Under allergist supervision, SDD was performed once the index reaction was controlled. At a later date, clinical phenotypes and endotypes of DHRs were assessed through clinical history, skin tests, serum biomarkers (including tryptase and IL-6 levels), and drug provocation testing (DPT) to reach an accurate diagnosis.

**Results:**

SDD was successful in 35 cases, even for patients with severe initial reactions. Only 14% experienced breakthrough reactions, all mild. Same-day assessment by allergists ensured a 92% correlation between initial and final diagnoses, optimizing DHR management. Early engagement with Allergy allowed 86% of reactive patients to continue treatment through RDD or after ruling out an allergy.

**Conclusion:**

SDD is a safe and effective procedure that ensures that patients don't miss their oncology treatment on the day of a reaction. The presence of an allergist in the OIC is crucial for rapid access to accurate DHR phenotyping and optimal management, supporting personalized precision medicine in oncology.

## Introduction

### Background

In recent years, the advent of precision oncology has transformed cancer treatment through advancements in tumor biology and the development of new antineoplastic drugs. This evolution has significantly improved outcomes for oncology patients, with survival rates doubling compared to traditional therapies (1, 2). However, this pharmacological progress is accompanied by treatment toxicities, notably DHRs (3). When oncologic patients experience DHRs leading to treatment interruptions, they are likely to face reduced survival rates (4). This disruption adversely affects their overall clinical outcomes and heightens their emotional burden (5).

### Current challenges

Drug hypersensitivity reactions are heterogeneous and complex, necessitating precise phenotyping and endotyping for effective management (6). Timely diagnosis and management are crucial for improving outcomes in patients affected by hypersensitivity reactions to antineoplastic and biologic drugs. Interdisciplinary collaboration between Oncology and Allergy is essential for accurate diagnosis and optimal treatment (7–9). Properly addressing the complexities of DHR management in cancer therapy is impossible without the synergistic efforts of oncology and allergy specialists (4).

### Rapid drug desensitization (RDD)

Rapid Drug Desensitization (RDD) is a well-established procedure that allows the safe re-administration of antineoplastic or biologic drugs after a patient has experienced an DHR (10–12). The RDD process is conducted in a highly controlled environment, utilizing a multi-step protocol that gradually increases the drug dose by adjusting rate flow, concentration or volume (13). Different research groups have published various RDD protocols; the protocol choice is based on the patient's allergological profile, risk stratification, and local needs (4, 14–19). Notably, when administered by an allergy-led team of expert allergists with appropriate resources and facilities, patients receiving oxaliplatin and carboplatin via RDD showed survival outcomes comparable to those receiving standard treatment (20, 21).

### Introduction of Same-Day Desensitization (SDD)

We recently introduced a novel type of RDD procedure called Same-Day Desensitization (SDD) to enhance patient quality of life and mitigate the emotional burden associated with treatment delays or interruptions following DHRs (7). SDD facilitates the continuation of drug administration on the same day as the patient's reaction, thus preventing treatment delays while maintaining therapeutic schedules (5, 7, 22).

SDD involves restarting the chemotherapy drug infusion that triggered the DHR, using a specific protocol, once the reaction has been adequately treated and controlled (7). This approach takes advantage of the cellular refractory period, known as post-anaphylactic mast cell anergy, or “mast cell emptying syndrome”, allowing the reintroduction of the drug without triggering another reaction (23). The total therapeutic dose is then administered, ensuring treatment continuity on the same day, preventing delays and maintaining therapeutic schedules (7).

Successful implementation of SDD requires a multidisciplinary team, including highly qualified personnel and the presence of an allergist in the OIC. Following SDD, patients are referred for allergy consultations to investigate further and confirm the phenotype of the DHR (7).

While SDD offers significant benefits, one limitation is the initial treatment of patients experiencing DHRs without definitive knowledge of the underlying mechanisms or exact diagnosis. The characterization of DHRs and their phenotyping typically occurs in a subsequent phase, utilizing standard *in vivo* and *in vitro* biomarkers (6). Nevertheless, the presence of an allergist in the OIC enables immediate treatment of the initial reaction and the establishment of desensitization protocols, alongside achieving a correct diagnosis based on clinical markers.

This study aims to analyze the diagnostic capacity of the allergist in an OIC following a DHR, relying on witnessed clinical history and biomarkers. During the SDD process, no additional test results are available to substantiate the definitive diagnosis; thus, SDD is executed based on the phenotype inferred from the patient's clinical presentation and the implicated drug's characteristics. The allergist's involvement at the OIC facilitates the initial diagnosis and treatment of infusion reactions in oncology patients experiencing hypersensitivity reactions to antineoplastic agents.

### Objectives of the study

• Primary: To evaluate the safety and efficacy of SDD in patients experiencing hypersensitivity reactions to chemotherapy agents.
• Secondary: To determine the allergist's phenotyping accuracy post-SDD.
• Supplementary: To assess the outcomes of subsequent RDD procedures following SDD and to investigate the essential role of the allergist in actively collaborating with the OIC.

## Methods

### Ethics approval statement

The Castellon Provincial Hospital Consortium Ethics Committee approved the study protocol. All participants provided written informed consent to participate in the study.

### Study design and population

Retrospective cohort study involving patients who experienced immediate hypersensitivity reactions to antineoplastic or biologic agents at the OIC between February 2021 and July 2022. Patients were distributed by sex, mean age, and neoplastic disease diagnosis.

### Initial reaction assessment

Initial reactions were classified into Type I immediate reactions (IgE-mediated or non-IgE-mediated), cytokine release reactions (CRR), infusion related-reactions and mixed reactions (19). Currently, infusion-related reactions are considered a subtype within DHR, and they are clinically like the mildest phenotype CRR (24). Infusion-related reactions are self-limited, do not require desensitization and can be managed by decreasing the rate of infusion of the original medication with or without premedication (19, 25).

The severity of Type I immediate reactions was graded using Brown's severity scale (1, 2, and 3) (26).

Cytokine release reactions were graded based on the Common Terminology Criteria for Adverse Events (CTCAE) from the National Cancer Institute (NCI) (27). Patients were further categorized by the culprit drug (oxaliplatin, carboplatin, paclitaxel, doxorubicin, and cetuximab) and whether it was an initial treatment or re-treatment (patients who, after a disease-free period, are given the same drug they were initially treated with again).

A recent communication proposed the existence of a “converting phenotype”. This phenotype was described in patients treated with taxanes who initially presented with non inmediate drug hypersensitivity reaction (NIDHR) and who, after subsequent exposures, developed inmediate drug hypersensitivity reaction (IDHR), generally Type I hypersensitivity (28). It is now proposed that this phenotype is not unique to taxanes (29).

#### SDD candidate patients

Patients were considered eligible for SDD if they met the following inclusion criteria:

1. DHR during drug infusion.
2. Clinical presentation was witnessed and treated by an allergist.
3. Oncologist-confirmed necessity for continued treatment with the culprit drug.
4. Clinical stabilization after treatment.
5. Signed informed consent.

Patients not meeting the SDD criteria (criteria 3, 4, or 5) were excluded from the SDD protocol and referred to the Allergy Department for further evaluation.

Patients who had uncontrolled bronchial asthma or uncontrolled cardiac disease, were suspected of having severe immunocytotoxic reactions (e.g oxaliplatin-induced immune thrombocytopenia) and/or, after appropriate treatment, did not achieve haemodynamic stability, were excluded from the SDD procedure.

#### SDD protocol

The SDD protocol, designed to last approximately 3.6–4 h, used a single drug dilution over 10 steps (Table 1) (7). Dose increments of 2- to 2.5-fold were administered at 15-minute intervals. Based on previous experience with one bag-RDD, the protocol was flexible and adjusted based on the severity of the initial reaction and patient response (17, 18).

**Table 1:** Example of the protocol used for the Same-Day Desensitization, using a non-diluted 1 bag/10 step protocol (7). Example of dosing for 100 mg of oxaliplatin.

	• Concentrate solution for infusion contains:				**5 mg/ml**
	• Total dose of oxaliplatin:				**100 mg (20 ml)**
	• Total volume in the bag: 250 ml glucose solution + Oxaliplatin dose:				**270 ml**
	• Normal concentration of the bag: 100 mg/270 ml:				**0.37 mg/ml**
	**Example when the reaction appears at 40 ml of volume infused:**				**14.8 mg (40 ml)**
	• Remaining dose to be administered after the reaction:				**85.2 mg (230 ml)**
STEP	Rate ml/hour	Time (min)	Administered volumen (ml)	Administered dose (mg)	Cumulative dose infused (mg)
1	0.6	15	0.15	0.06	0.06
2	1.2	15	0.3	0.11	0.17
3	2.4	15	0.6	0.22	0.39
4	4.8	15	1.2	0.44	0.83
5	9.6	15	2.4	0.89	1.72
6	19.2	15	4.8	1.78	3.50
7	38.4	15	9.6	3.56	7.06
8	76.8	15	19.2	7.11	14.17
9	100	15	25	9.26	23.43
10	120	83.37	166.75	61.76	85.19
	**TOTAL (SDD)**	**218** **.** **4**	**230**	**85** **.** **20**	**85** **.** **20**
				
	**Previous to reaction:**		**40 ml**	**14.8 mg**	
	**TOTAL (Administered)**		**270 ml**	**100 mg**	

mg, milligrams; mg/ml, milligrams/millilitres; ml/h, millilitres/hour; min, minutes; SDD, Same Day Desensitization.

This process capitalizes on the mechanisms of RDD, which gradually increases the drug concentration and exploits the unique physiology of mast cells (4). Slowly raising the ligand's dose prevents mast cells from becoming activated, thus avoiding the release of mediators responsible for hypersensitivity reactions by blocking key steps in mast cell activation, such as calcium influx, degranulation, and the production of lipid mediators and cytokines (4, 30).

In addition, SDD utilizes the refractory state of degranulated mast cells, potentially related to post-anaphylactic mast cell anergy or “mast cell emptying syndrome” (23).

#### SDD premedication and concomitant drugs

Patients received standard premedication for the implicated chemotherapy agent as per the manufacturer's information and institutional protocols. Prior to SDD, patients also received acute treatments to resolve the initial DHR. For patients on beta-blockers, alternative treatments such as glucagon were made available in case of epinephrine ineffectiveness during anaphylaxis (31). Following SDD, patients received their additional prescribed oncologic treatments as usual.

#### SDD location

All SDDs were performed in the OIC, which was equipped with facilities including trained nursing staff, continuous allergist presence, hazardous drug handling measures, constant patient monitoring, piped oxygen, crash cart, and rapid access to intensive care for emergency management.

#### Breakthrough reactions during SDD

**Figure 1:**
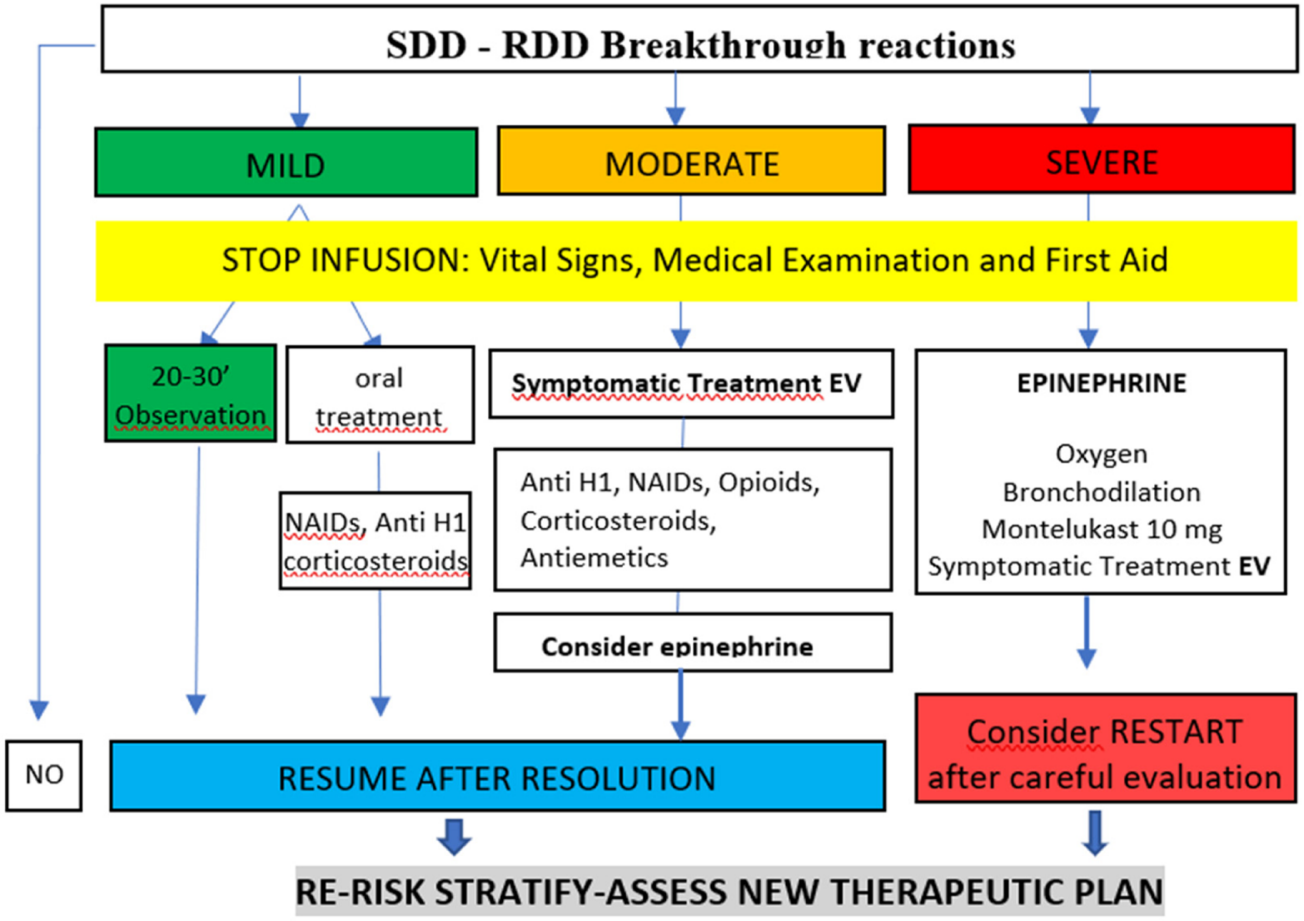
Management of the breakthrough reactions (BTR) during SSD-RDD. Adapted from Vega et al. (32).

Breakthrough reactions (BTRs) during SDD were managed similarly to those occurring during RDD (Figure 1) (32, 33). The average time to resume drug administration after a BTR is around 20 min, depending on the severity of the BTR and the patient's response to treatment (7).

#### Diagnostic protocol: skin test, serological biomarkers and drug provocation test (DPT)

After SDD, patients were referred to the Allergy Department for further allergological studies. Skin prick tests (SPT) and intradermal tests (IDT) were performed following international guidelines (34). These tests were conducted for all implicated drugs except doxorubicin due to its vesicant nature Table 2 (35, 36). Skin tests were performed two weeks after the initial reaction to avoid false negatives, except in specific cases due to clinical needs (weekly therapeutic regimens) (37).

**Table 2:** Concentrations used for SPT and IDT (35, 36).

Drug	SPT	IDT
Paclitaxel	1/10: 0.1–0.6 mg/ml	1/1,000: 0.001–0.006 mg/ml
6 mg/ml	1/1: 1–6 mg/ml	1/100: 0.01–0.06 mg/ml
		1/10: 0.6 mg/ml
Carboplatin	1/1: 10 mg/ml	1/100: 0.1 mg
10 mg/ml		1/10: 1 mg
Oxaliplatin	1/1: 5 mg/ml	1/100: 0.05 mg
5 mg/ml		1/10: 0.5 mg
		1/1: 5 mg
Cetuximab	1/1: 5 mg/ml	1/10: 0.5 mg/ml
5 mg/ml		1/1: 5 mg/ml
Doxorubicin	Not performed	Not performed

Serological biomarkers, including total IgE (ImmunoCAP™ Total IgE. Uppsala, Sweden), interleukin-6 (IL-6, human IL-6—Immunoassay Quantikine® ELISA. Minneapolis, USA), and tryptase (ImmunoCAP™ Tryptase. Uppsala, Sweden), were measured post-reaction (90 min after DHR onset) and baseline (two weeks after DHR) (37). Patients with negative or inconclusive skin tests or serological results and a favorable risk assessment underwent a DPT.

#### Allergist´s role and subsequent RDD

The initial suspected phenotype prior to SDD was confirmed or revised after the allergological workup. We also recorded the number of subsequent RDD procedures performed after SDD and whether any breakthrough reactions occurred during these later procedures.

## Results

### Patient characteristics

A total of 35 patients participated in the study, with 17 (49%) females and 18 (51%) males.

The mean age was 58 years (range: 37–79 years). Diagnoses included colorectal cancer (51%, *n* = 18), gastric cancer (23%, *n* = 8), ovarian cancer (11%, *n* = 4), breast cancer (3%, *n* = 1), lung cancer (3%, *n* = 1), esophageal cancer (3%, *n* = 1), pancreatic cancer (3%, *n* = 1), and urologic cancer (3%, *n* = 1).

The most frequently implicated drugs were oxaliplatin, carboplatin and paclitaxel, with doxorubicin and cetuximab also noted (Table 3).

**Table 3:** Demographic and clinical characteristics of the patients.

	Total *n* = 35	
Sex		
Male	18	(51%)
Female	17	(49%)
Age		
Average	58	
Range	37–79	
Diagnosis		
Colorectal	18	(51%)
Gastric	8	(23%)
Ovarian	4	(11%)
Breast	1	(3%)
Lung	1	(3%)
Esophagus	1	(3%)
Pancreas	1	(3%)
Urologic	1	(3%)
TNM staging		
T2	2	(6%)
T3	11	(31%)
T4	22	(63%)
Associated mutations		
BRCA	0	
BRAF	6	
RAS	2	
HER2	2	
Drug		
Oxaliplatin	28	(80%)
Carboplatin	3	(9%)
Paclitaxel	2	(6%)
Doxorubicin	1	(3%)
Cetuximab	1	(3%)
Cycle in which the reaction was presented		
Average	4	
Range	1–12	
Periordicity of cycles		
Weekly (7)	1	(3%)
Every 15 days	13	(37%)
Every 21 days	20	(57%)
Every 28 days	1	(3%)
Retreatment		
Yes	24	(69%)
No	11	(31%)
Volume infused (in the initial HR)		
Average	76.57	ml
Range	7–256	ml
Type of infusion (according to oncological prescription)		
Normal	13	(37%)
Slow	22	(63%)
Suspected phenotype		
Type 1 reaction	31	(88%)
Infusional reaction	2	(6%)
Cytokine release reaction	0	
Mixed reaction	2	(6%)
Final diagnosis		
Type 1 reaction	27	(77%)
Infusional reaction	5	(14%)
Cytokine release reaction	0	
Mixted reaction	3	(9%)

### Initial reactions details

According to the initial reaction phenotype, 31 patients (88%) had Type I immediate reactions, 2 patients (6%) experienced infusion related-reactions, 2 patients (6%) presented mixed reactions and no patient had symptoms compatible with CRR phenotype exclusively.

Among the 31 patients with type I reactions, 23 were grade 1, 7 were grade 2, and 1 was grade 3. For mixed reactions and infusion related-reactions, two patients presented with grade 1 reactions, and two with grade 2 reactions (Table 4).

**Table 4:** Clinical presentation and severity according to drug.

	Oxaliplatin (*n* = 28)	Carboplatin (*n* = 3)	Paclitaxel (*n* = 2)	Doxorubicin (*n* = 1)	Cetuximab (*n* = 1)
Symptomatology					
Shivering	1	0	0	0	0
Hypertension	4	0	2	0	0
Flushing	5	0	1	0	0
Cutaneous	22	3	1	1	1
Digestive	6	1	0	0	1
Respiratory	4	2	0	1	1
Cardiac	0	1	0	1	0
Hemodynamic inestability^a^	2	0	1	1	1
Severity according to Brown's scale					
Grade 1	21 (75%)	1 (33%)	1 (50%)		
Grade 2	5 (18%)	2 (67%)			
Grade 3					1 (100%)
Severity according to NCI					
Grade 1	2 (7%)				
Grade 2			1 (50%)	1 (100%)	
Grade 3					
Grade 4					
Grade 5					

^a^ Hemodynamic instability including desaturation, hypotension or tachycardia.

The two patients who experienced an infusion-related reaction were not re-exposed to the drug on the same day of the reaction. Both received the remaining drug through SDD because, at that time, they did not have biomarkers that would allow us to rule out a more serious reaction, such as a CRR.

### Efficacy of Same-Day Desensitization (SDD)

The SDD protocol, which lasted approximately 3.6–4 h, successfully desensitized all patients. During SDD, 14% of patients experienced breakthrough reactions (BTR), all classified as mild (Brown grade 1, CTCAE-NCI grade 1). All patients with BTRs responded well to treatment and completed the procedure without further incidents.

The majority of patients (77%) had a positive skin test, while 14% had a negative result. In 9% of cases, skin tests were not performed due to various circumstances. Among those with positive skin tests: 11% were positive in skin prick tests (SPT), 29% were positive in intradermal tests (IDT) at 1/100 concentration, 31% were positive in IDT at 1/10, and 6% were positive in IDT at 1/1 (Table 5).

**Table 5:** Allergological explorations.

Positive skin tests	*N* = 35	
Prick	4	(11%)
ID 1/100	10	(29%)
ID 1/10	11	(31%)
ID 1/1	2	(6%)
Negative	5	(14%)
Not performed	3	(9%)
Post reaction tryptase (ng/ml)		
Average	8.5	
Range	1–39.8	
Basal tryptase (ng/ml)		
Average	5.9	
Range	2.3–11.9	
Post reaction IL-6 (pg/ml)		
Average	93	
Range	1.2–2,370	
IL-6 Basal (pg/ml)		
Average	14.25	
Range	0.9–61	
IgE total (IU/ml)		
Average	418	
Range	2.8–3,891	

The mean post-reaction tryptase was 8.5 ng/ml (range: 1–39.8 ng/ml), while the mean IL-6 level was 93.15 pg/ml (range: 1.2–2,370 pg/ml). The mean total IgE level was 418 IU/ml (Table 5).

### Diagnostic outcome

The initial suspected phenotype prior to SDD was confirmed in 92% of patients (32 patients) after the allergological study.

Three patients, in whom the suspected phenotype prior to SDD was a Type I reaction, were finally diagnosed as having an infusion-related reaction after allergology study. All three patients had negative skin tests with oxaliplatin, paclitaxel and cetuximab respectively. One of the patients, after a negative DPT, continued with normal infusions by making adjustments to the infusion rate. In the other two patients, DPT did not perform with the culprit drug because the oncologist decided to change the line of treatment for clinical reasons.

Two patients initially phenotyped as Type I reaction were later confirmed as having mixed reactions (Type I and cytokine release). One patient exhibited symptoms of cytokine release 45 min after SDD, with post-reaction IL-6 at 219.6 pg/ml. The final diagnosis was a mixed reaction, allowing the patient to continue receiving oxaliplatin via RDD. The other patient developed symptoms of cytokine release 45 min post-drug provocation test, with a final diagnosis confirming the mixed phenotype. This patient continued with the administration of the drug through RDD.

### Management of breakthrough reactions (BTR)

BTR occurred in 14% of patients during SDD, all mild and managed effectively. Treatments included intravenous antihistamine (dexchlorpheniramine 5 mg) for 3 patients (9%), intravenous corticosteroids (methylprednisolone 1–2 mg/kg or hydrocortisone 100–500 mg) for another 3 patients (9%), and a combination of both for 27 patients (77%). Additionally, 2 patients (6%) required intravenous antihistamine, corticosteroids, and intramuscular epinephrine (0.5 mg).

After SDD, all patients were observed for one hour before discharge, except for one patient who showed clinical signs of cytokine release and required 24 h of observation. In cases with severe initial DHR or BTR during SDD, oral antihistamines were prescribed for home use over 48 h.

### Subsequent rapid drug desensitization (RDD) procedures

Following SDD, 29 patients (83%) continued their chemotherapy regimen with RDD procedures. A total of 118 RDDs were performed on a scheduled basis. Of these, 107 RDDs occurred without incident, while 11 experienced BTR during the procedure. No patients developed a converting phenotype in subsequent RDD procedures.

## Discussion

### Effectiveness and safety of Same-Day Desensitization (SDD)

Our study confirms that SDD is both a safe and effective intervention for patients experiencing hypersensitivity reactions during drug administration. Notably, all patients undergoing SDD successfully and safely received the total prescribed dose, including those experiencing BTRs, as all were mild. The data indicates that SDD does not increase the risks associated with DHRs, even among patients with initial severe reactions (grade 3 Brown and grade 2 CTCAE-NCI). This finding supports the assertion that SDD does not pose a greater risk than suspending treatment on the day of the reaction and waiting for programmed or elective RDD later.

Current literature includes only one prior publication detailing the SDD procedure (7). While RDD remains the cornerstone for managing reactions to chemotherapy and biologics, it presents challenges, such as the potential loss of the treatment cycle during which the DHR occurs due to delays in allergy workup and scheduling RDD for subsequent administrations. SDD effectively addresses this issue by ensuring that the patient does not miss the treatment on the day of the index reaction (7).

The most significant advantage of SDD is its ability to maintain the first-line therapeutic regimen in oncology patients, thereby preventing losses or delays in treatment administration, which has both clinical and emotional benefits for the patient. Moreover, SDD is more cost-effective, as it prevents medication waste and ensures adherence to the therapeutic schedule established by the oncologist, a crucial factor for patient survival (5, 9). Additionally, early engagement with the allergist alleviates patient anxiety by minimizing treatment cancellations and providing reassurance.

### Insights from previous experiences

Based on our previous published experience with SDD initiated at step 1 for nine patients with excellent outcomes, the current study initiated SDD in most patients at step 4 or 5 of the 1/1 bag (Table 1) (7). However, caution should be exercised for highly reactive patients, particularly those reacting to platinum salts, who, lacking better data, may require initiation of the SDD procedure with a 1/100 bag (4 steps) before progressing to the initial bag with 10 steps.

### The role of allergology diagnosis and phenotyping

This work illustrates the critical role of allergists in the OIC for the effective management of DHRs. The preliminary phenotyping of the initial reaction is particularly relevant, as it aids in predicting desensitization outcomes and tailoring future treatment plans (38).

Among the patients in our study, 92% had their suspected phenotypes confirmed following SDD, indicating high pre-SDD phenotyping accuracy by expert allergists and an excellent completion rate for allergy workup. Only three patients, initially diagnosed with a type I reaction, could not have their phenotype confirmed after the allergy workup.

These 3 patients received their full treatment on the same day of the reaction using SDD. All three patients had negative skin tests with the suspected drug and non-significant tryptase levels after the reaction. The drugs involved were paclitaxel, oxaliplatin and cetuximab. One of the patients, after negative PTLD, continued with regular infusions with rate adjustments without further incidents. In the other two patients, DPT was not performed because their oncology treatment ended after SDD for clinical reasons and therefore they could not be re-exposed.

Prior studies indicate that nearly 40% of patients with initial type I hypersensitivity reactions to oxaliplatin converted to other endophenotypes (most commonly complex regional reactions or mixed reactions) during RDD (38). This may explain the two cases later diagnosed with mixed reactions to oxaliplatin. The rapid recognition of symptoms by the allergist present at the OIC could prevent the progression of incipient type I reactions to more severe manifestations during initial reactions before SDD.

Our findings related to subsequent RDD outcomes suggest that while a positive skin test, especially a positive SPT, significantly predicts reactions during desensitization, the allergist's involvement facilitates accurate diagnosis and necessary evaluations during DHR events.

### Serum biomarkers and clinical indicators

Previous publications convey that tryptase determination during the acute phase of DHR is useful for confirming mast cell involvement, with higher tryptase values associated with more severe drug reactions (6, 39). However, we observed that patients with low clinical severity (exclusively cutaneous) could have significantly elevated post-reaction tryptase levels. These results highlight that post-reaction serum tryptase levels are not always directly related to clinical severity. Future research should clarify whether this discrepancy may be linked to the allergist's prompt action at the OIC at the onset of the reaction.

Previous publications have found that an average elevation of about 40 times the normal serum IL-6 concentration helps define the oxaliplatin CRR endophenotype (38, 40). We observed that IL-6 levels below 50 pg/ml are not typically correlated with clinical cytokine release. All patients presenting clinical symptoms compatible with cytokine release had IL-6 levels equal to or exceeding 100 pg/ml, confirming its utility as a biomarker for identifying CRR.

To accurately diagnose patients, we must consider a series of biomarkers whose results may be unknown when SDD is initiated immediately after the initial DHR. However, we should not underestimate the importance of clinical markers for later endophenotyping an DHR, reinforcing the necessity of allergist involvement in the OIC.

It is also essential to recognize that different endotypes may coexist in the same patient, potentially inducing reactions via various mechanisms, such as immunologically mediated (IgE or IgG) or non-immunologically mediated mechanisms (e.g., mast cell activation related to G protein-coupled receptor X2). This may lead to a synergistic rather than exclusionary effect (6, 39, 41).

### Results of RDD

After SDD and allergy workup, 86% of patients could continue their treatments either by RDD or after a negative DPT. Twenty-nine patients underwent 118 RDD under allergy care, which is an average of 4 procedures per patient. We observed a total of 11 BTRs in 5 patients among the 118 RDD procedures performed (9% of BTRs). The severity of BTRs according to Brown's classification were grade 1 (6/11), grade 2 (5/11) and grade 3 (0/11). That is, 55% of the BTRs were mild, 36% were moderate and 0% were severe. They all received their target treatments, and safety profile was like previous publications (12).

Some studies indicate that a positive skin test result (especially a positive SPT) is a crucial predictor of reactions during desensitization, as corroborated by our findings, where all patients who experienced breakthrough reactions during subsequent RDD had positive skin tests (12, 33). 60% of patients who experienced BTR during RDD had a positive SPT while 40% of patients who experienced BTR during RDD had a positive IDT 1/100.

### The essential role of the allergologist

The presence of the allergist in the Oncology Infusion Center (OIC) facilitates rapid and accurate diagnosis of patients experiencing hypersensitivity reactions, ensures timely biomarker collection, and allows for appropriate management strategies that may reduce the severity and duration of reactions. This engagement guarantees that the initial reaction is thoroughly evaluated, aligning the phenotype closely with the diagnosis. Furthermore, comprehensive assessments conducted during the hypersensitivity reactions occurrence enhance patient care and alleviate anxiety, contributing to a more supportive environment for patients during critical treatment phases. Notably, the immediate access to the allergy team enables these patients to benefit from the effective technique of SDD, further optimizing their treatment experience. There is also a pressing need for allergists to take responsibility for their leading role in multidisciplinary collaboration in managing DHRs to enhance patient outcomes (4).

### Limitations and future directions

Our study acknowledges certain limitations, including its retrospective design and unicentric nature, which raise questions about the universality of SDD, especially as local variations in practice may influence how each center implements this approach.

Other teams have successfully employed “restart protocols” immediately following DHRs, where they simply restart the drug during reactive drug provocation tests (DPT) with excellent results, without the need for a SDD (12).

This could suggest that desensitization may be unnecessary in some cases, possibly because post-anaphylactic mast cell anergy applies. However, it is important to note that the experiences of those authors were conducted in the context of DPT performed in an intensive care unit, not in the OIC. Hence, our protocol aims to utilize various mechanisms—not only relying on the concept of post-anaphylactic mast cell anergy but also incorporating RDD—to maximize safety and tolerance during such critical moments for the patient.

Future research should focus on refining SDD protocols, exploring implementation in other cohorts, and determining which quality indicators improve when expert allergists lead in the care of these patients.

## Conclusion

In summary, our findings highlight the effectiveness and safety of Same-Day Desensitization (SDD), enabling patients to complete their prescribed treatment without delays. The involvement of allergists in the Oncology Infusion Center (OIC) is essential not only for the success of SDD but also for the optimal assessment and management of acute drugs hypersensitivity reactions. Our study demonstrates a high degree of diagnostic concordance, reinforcing the allergist's pivotal role in accurate DHR phenotyping and desensitization. Accurate phenotyping is crucial for effective risk stratification and the development of RDD protocols within personalized medicine. Therefore, we advocate for increased resources and the establishment of allergy-led multidisciplinary teams to integrate these techniques as fundamental components of comprehensive oncology care.

## Data Availability

The raw data supporting the conclusions of this article will be made available by the authors, without undue reservation.
